# ASSOCIATIONS BETWEEN CUMULATIVE STRESS AND COGNITIVE FUNCTION IN HRS AND ELSA

**DOI:** 10.1093/geroni/igac059.1500

**Published:** 2022-12-20

**Authors:** Ruijia Chen, Harold Lee, Sakurako Okuzono, Laura Kubzansky

**Affiliations:** University of California, San Francisco, San Francisco, California, United States; University Of North Carolina At Chapel Hill, Chapel Hill, North Carolina, United States; Department of Social and Behavioral Sciences, Harvard T.H. Chan School of Public Health, Boston, Massachusetts, United States; Department of Social and Behavioral Sciences, Harvard T.H. Chan School of Public Health, Boston, Massachusetts, United States

## Abstract

Despite growing research linking stressors with poorer cognition, less research explicitly considers stress from several sources, which often co-occurs and accumulate to influence health. We used the Global Gateway Harmonized data (HRS: n=8,888; mean age: 74 years; ELSA: n=6,715; mean age: 65 years) to examine whether higher cumulative stress is associated with lower levels of and faster decline in cognitive function. We fit linear mixed effect models to assess the stress-cognition associations. After adjusting for all covariates, baseline cumulative stress was associated with lower baseline cognitive function in both HRS (β=-0.02, 95%CI -0.04, -0.01) and ELSA (β=-0.03, 95%CI -0.05, -0.01). Unexpectedly, higher baseline cumulative stress was associated with slower cognitive decline in HRS (βtime*stress=0.001, 95%CI 0.002,0.01) but not in ELSA. The stress-cognition associations may differ among adults in the US and the UK. Future research should investigate how cumulative stress may operate differently to influence cognitive function across different populations.

